# Handheld Osteotome Laminectomy in Cadavers and a Case Study

**DOI:** 10.7759/cureus.53867

**Published:** 2024-02-08

**Authors:** Alice S Wang, Paras Savla, Saman Farr, Farbod Asgarzadie, Dan E Miulli

**Affiliations:** 1 Neurosurgery, Riverside University Health System Medical Center, Moreno Valley, USA; 2 Neurosurgery, Kaiser Permanente Fontana Medical Center, Fontana, USA; 3 Neurosurgery, Arrowhead Regional Medical Center, Colton, USA

**Keywords:** surgery, spine, case study, laminectomy, osteotome

## Abstract

Laminectomy is a commonly performed surgery to decompress the spinal canal to relieve spinal canal stenosis secondary to a variety of etiologies such as degenerative spinal changes, fractures, tumors, vascular lesions, and infections. Advances in technologies have allowed for more precise osteotomies and offer more protection to nearby structures; however, these technologies may not always be available at some facilities. To the best of the authors' knowledge, we describe an innovative technique to perform laminectomy using a handheld osteotome, which is widely available and at low cost. Our experience with cadavers and a case study shows that the technique appears to be safe and effective and may have the potential to reduce the procedure length of a laminectomy.

## Introduction

The first successful laminectomy was documented in 1838, although the first laminectomy was performed in 1814 [1]. Since then, decompressive laminectomies have become commonly performed surgeries. The main goal of a laminectomy is to decompress the spinal canal to relieve spinal canal stenosis. Spinal canal stenosis can be secondary to degenerative stenosis, fractures, spinal tumors, spinal vascular lesions, epidural abscesses, and congenital deformity [2]. Indications for laminectomy may include patients refractory to non-surgical management, such as medication, physical therapy, and epidural injections; presence of intractable pain or progressive neurological deficits; and cauda equina syndrome [2,3]. Laminectomy can be achieved with open or minimally invasive approaches. Various technological advances, including high-speed drills and ultrasonic osteotomes, have allowed the surgeon to make precise osteotomies while protecting adjacent soft tissue structures [4].

Haddas, et al. demonstrated a technique using an ultrasonic osteotome in a retrospective study of 85 patients who underwent the H laminectomy. In this technique, two longitudinal troughs were made from the top to the bottom of the laminae of interest, followed by a transverse trough through the superior third of the lamina, connecting the longitudinal troughs, forming the letter H. Then, the superior third of the lamina was removed using rongeurs. They did not cause any dural tears or cerebrospinal fluid (CSF) leaks. The constant visualization and tactile feedback of the tip of the instrument and osteotome provided an inherent safety mechanism and allowed enhanced control of the dura-ligamentum interface, even for non-experienced surgeons. They concluded that the H laminectomy is a safe and effective way to perform a lumbar laminectomy [5].

While technological advances such as the ultrasonic osteotome have been helpful in performing laminectomies, some facilities may not have these tools due to cost or may not be familiar with how to use them to perform laminectomy. On the other hand, a handheld osteotome is widely available and at low cost. We describe using a handheld osteotome to perform a decompressive laminectomy at our institution, a technique that, to our knowledge, has not been described in the literature.

Experience in cadavers 

**Figure 1:**
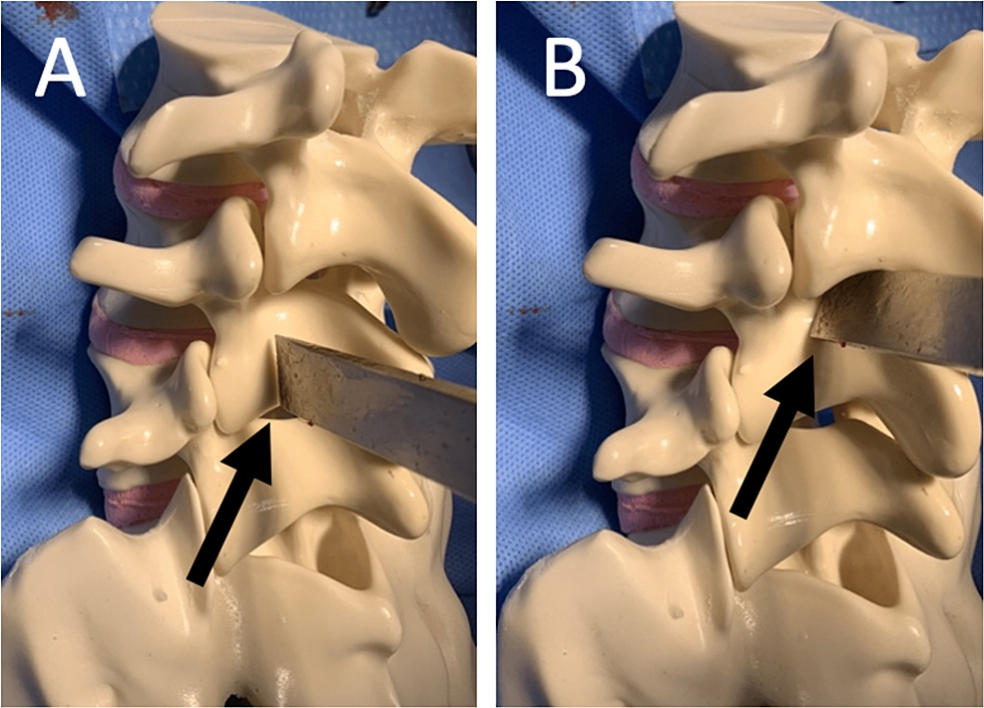
Osteotome on lamina and pars interarticularis (A) The osteotome is positioned at the junction of lamina and facet to make the vertical cut, one on each side of the interested level. The black arrow points to the tip of the osteotome. (B) The osteotome is positioned at the pars interarticularis to make the oblique cut, one on each side of the interested level. The black arrow points to the tip of the osteotome.

**Figure 2:**
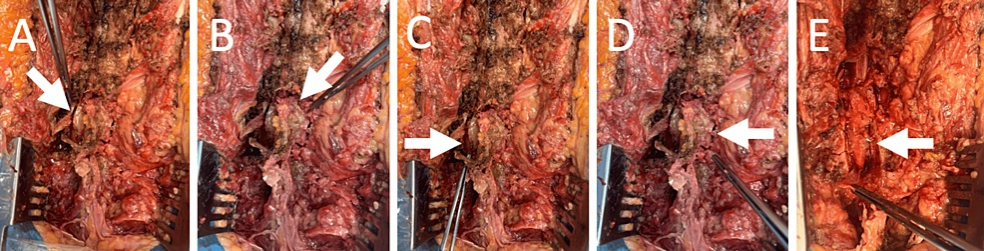
Handheld osteotome laminectomy in a cadaver at our institution In a cadaver, the forceps indicate the oblique cuts at the left pars interarticularis in (A) and at the right pars interarticularis in (B). The white arrow in (A) highlights the left oblique cut, and the white arrow in (B) highlights the right oblique cut. The forceps identify the vertical cuts at the left lamina in (C) and at the right lamina in (D). The white arrow in (C) points to the left vertical cut, and the white arrow in (D) points to the right vertical cut. (E) displays the forceps reflecting the ligamentum flavum to expose the epidural fat and dura, which remain intact. The white arrow in (E) indicates the dura.

Our technique is similar to the Smith-Peterson osteotomy, an approach for flexion deformity correction where bilateral facet joints, partial lamina, and posterior ligaments are resected at the osteotomy site to bring the posterior column closer and open the anterior column [6]. Here, we first describe our experience in cadavers where laminectomies from lumbar one to five were performed. A posterior midline lumbar incision was made down to subcutaneous fat followed by a subperiosteal dissection. Then, the superior and inferior interspinous ligaments at the level of interest were divided with a Bovie. A spine navigation system was utilized, placing the frame on the spinous process at the level below. We left the spinous process on the lamina of interest in place so that Kocher forceps could be used to grasp and remove the lamina. The lamina and the pars interarticularis were identified. Using a handheld osteotome and mallet, two vertical cuts were planned with the spinal navigation system and made on the lamina, one on each side at the level of interest. Next, two oblique cuts were made at the pars interarticularis, one on each side at the level of interest (Figures 1, 2). At this point, the lamina was freed from bony attachments. Next, a Kocher was used to grasp the spinous process and gently elevate the lamina, which was attached to the underlying ligamentum flavum, epidural fat, and dura. A Woodson elevator and upgoing curette were used to detach these structures from the lamina. The lamina was then freely removed with the Kocher. The ligamentum flavum, epidural fat, and dura were visualized and appeared to remain intact. A deficiency in cadaver was a lack of CSF distended dura and spinal stenosis. This procedure was then repeated at an additional level, first making a partial cut with the osteotome, followed by confirmation with the spinal navigation system to ensure correct placement. Finally, osteotomes were used to complete the cuts, exposing and removing the lamina and then the ligaments. These were inspected visually. The process was then repeated on an additional cadaver from lumbar one to lumbar five without spinal navigation. The technique without the use of spinal navigation was taught to other residents and attending physicians, and the procedure was repeated on two more cadavers in the lumbar regions, with visual inspections completed. There were no violations of the dura or nerve structures. Our experience with cadavers demonstrates that this technique is safe and effective in performing laminectomy.

## Case presentation

Figures 1, 2 depict the precise use of the osteotome during the laminectomy procedure.

Case presentation

**Figure 3:**
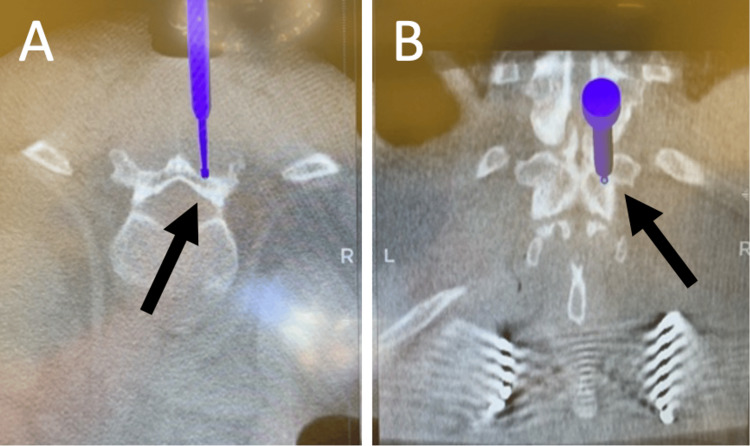
Intraoperative neuronavigation Intraoperative neuronavigation demonstrates the tip of the navigator probe is perpendicular at the junction between the lamina and the facet of the vertebral level of interest in the axial view in (A) and in the coronal view in (B). The black arrow in (A) indicates the right lamina in the axial view, and the black arrow in (B) indicates the right lamina in the coronal view.

**Figure 4:**
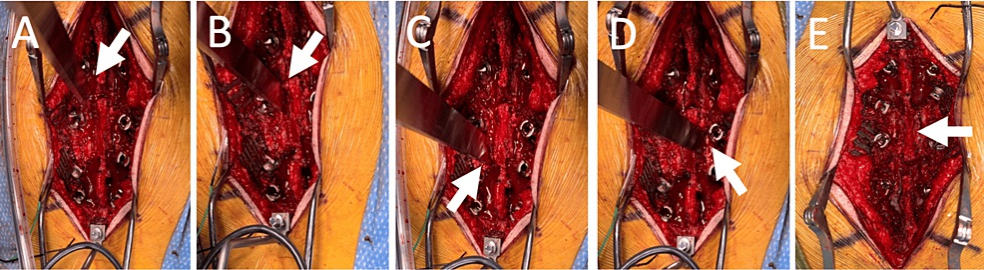
Handheld osteotome laminectomy in a case study In our case study, the osteotome makes an oblique cut at the left pars interarticularis in (A) and another one at the right pars interarticularis in (B). The white arrow in (A) indicates the left oblique cut, and the white arrow in (B) indicates the right oblique cut. The osteotome also makes a vertical cut at the left lamina in (C) and another one at the right lamina in (D). The white arrow in (C) points to the left vertical cut, and the white arrow in (D) points to the right vertical cut. After the lamina has been removed, (E) shows the dura remains intact, and there is no cerebrospinal fluid leakage. The white arrow in (E) highlights the intact dura.

A 27-year-old male presented with a complaint of back pain and inability to move his legs after landing on his buttocks from falling off a ladder approximately 8 feet from the ground. Prior to the injury, he was neurologically intact. After the fall, his motor strength was 5/5 in bilateral upper extremities, 1/5 in right hip flexion, and 0/5 in all other muscle groups in the lower extremities; sensory was intact at T12 level; and reflexes were positive for bulbocavernosus reflex, positive rectal tone, negative anal wink, and negative patellar and Achilles reflexes. Thoracic spine computed tomography (CT) demonstrated a T12 chance fracture with 50% loss of height, 25 degrees of angulation with associated T11 spinous process fracture, and 10 mm retropulsion with 60% canal encroachment. He was given an ASIA C Spinal Cord Injury score. The patient underwent open reduction and internal fixation (ORIF) of T12, T11-T12 laminectomy, and posterior lumbar arthrodesis from T10 to L2. Somatosensory evoked potentials (SSEPs), motor evoked potentials (MEPs), and electromyography (EMGs) were placed, then the patient was placed prone on the operating table. The patient was prepared and draped in the standard sterile fashion. Prophylactic antibiotics were given prior to incision. Monopolar cautery was used to dissect away the subcutaneous fat in the avascular plane. Subperiosteal dissection from the spinous process to the lamina to the transverse process was made using bipolar cautery from T10 to L2. A spinous process clamp was placed at L2. O-arm assisted navigation was used to localize the T12 fracture and the T10 to L2 vertebral segments were identified. Navigation was used for T10 to T12 pedicle screw placement. Next, T11-T12 laminectomies were performed. The interspinous ligaments of T10-T11 and T12-L1 were cut via rongeur. Next, after confirmation with the spinal navigation system, two vertical cuts were made on the lamina and two oblique cuts were made at the pars interarticularis at T11 and T12 to break through dorsal cortex and ventral cortex bilaterally (Figures 3, 4). A nerve hook was used to elevate the ligamentum flavum, which was resected bilaterally at the cranial and caudal margins via Kerrison rongeurs. A Kocher was placed at the T11 spinous process and the lamina was slowly elevated, freed from underlying dura with a Woodson elevator. There was no cerebrospinal fluid leakage. Two rods were placed and secured with cap screws. The wound was copiously irrigated with antibiotic irrigation. Decortication was carried out at the lamina, spinous process, and facet joints. A subfascial drain was placed. The incision was closed in the standard fashion. Postoperatively, the patient reported improved back pain. The motor exam improved to 4/5 in bilateral hip flexion, knee extension, and knee flexion, and 1/5 in dorsiflexion, plantar flexion, and hallucis extension. Sensory level remained intact around the T12 level. The patient was able to perceive proprioception and pressure touch in bilateral lower extremities. He was discharged to acute rehabilitation.

## Discussion

Decompressive laminectomies are common in spine surgery, benefiting from advancements like rongeurs, high-speed drills, and ultrasonic osteotomes, which provide precision and protect soft tissues [4]. Our technique involves two vertical cuts on the lamina and two oblique cuts at the pars interarticularis, differing from the H laminectomy method [5]. The cut across the lamina in the ultrasonic H laminectomy technique to free the lamina from its bony attachments can be dangerous because the spinal cord is directly underneath without the ligamentum flavum as a buffer. In our procedure, because we make the cuts at the pars interarticularis, which is safer, there is no need to cut across the lamina and run the risk of injury to the spinal cord. The ligament in this procedure is cut with scissors while the lamina is lifted off. The use of ultrasonic osteotomes has shown advantages over burrs, reducing operative time, blood loss, and study duration [7]. In a study by Hu et al., en bloc laminectomy using an ultrasonic osteotome shortened operative time from 94.8 to 65.9 minutes for the latter 75 of 151 patients [8]. While handheld osteotomes might raise concerns about feedback, our study suggests their safety in laminectomy procedures.

To mitigate risks during the laminectomy using a handheld osteotome, we meticulously identified key anatomical landmarks: spinous process, lamina, transverse process, facet joints, pars interarticularis, and interlaminar space. Proper placement of the osteotome at the lamina is crucial to avoid damage to the ligamentum flavum, spinal cord, or nerve roots during hammering. Positioning errors, such as medial or lateral misplacement, can lead to spinal cord or facet joint injuries. Careful targeting of the ventral cortex is essential; if too posterior, the lamina won't detach, and if too anterior, there's a risk of injuring exiting nerve roots. Perpendicular osteotome placement at the lamina-facet junction is necessary to prevent skiving or incomplete cutting. Elevating the lamina requires caution to avoid tearing the attached ligamentum flavum or dura, which could lead to cerebrospinal fluid leakage. Despite performing a pure laminectomy without O-arm navigation, our case study, which incorporated O-arm guidance for pedicle screw placement, helped verify anatomical landmarks and minimize risks. No spinal cord, nerve root, or CSF complications were encountered, and postoperative improvements were observed in motor, sensory, and reflex functions, with no changes in SSEPs, MEPs, and EMGs.

The use of a handheld osteotome to perform laminectomy provides several advantages. First, this technique is effective in removing the lamina, as demonstrated in the cadavers and our case study. Second, the technique appears to be safe, given the ligamentum flavum, epidural fat, and dura remain intact after the removal of the lamina. Third, this technique is thought to be fast relative to using a high-speed drill or ultrasonic osteotome, which the latter techniques can take a considerable amount of time in multi-level laminectomies. With a few simple motions with the mallet, the lamina can be freed of bony attachments. Prior to each hit with the hammer, we use either tactile feedback or navigation, if available, to ensure that the osteotome is on the lamina and not in an unsafe location. Fourth, a handheld osteotome is low-cost and widely available when compared to a high-speed drill or ultrasonic osteotome. Given that some facilities may not have advanced tools, the ability to use a handheld osteotome as a tool to perform laminectomy can be useful and powerful.

There are some limitations to our study as well. We document the experience with this novel method using multiple cadavers and a single intraoperative case study. Future studies should investigate if this technique is safe and effective in a large case series with various surgeons. The use of a handheld osteotome offers the potential to further reduce operative time, especially in multi-level laminectomies. Future studies should record the actual duration of laminectomies using different techniques. Also, our experience in the case study involved only one surgeon, and this calls for other surgeons to consider and perform this technique. Finally, technical advances from microscopes to, more recently, exoscopes have allowed for better intraoperative visualization of the surgical field through magnification and higher resolution. In a recent systematic review and meta-analysis comparing microscopes and exoscopes, the exoscope appears to be superior in video quality and offers better ergonomic features [9]. Future studies should investigate the role of the exoscope in handheld osteotome laminectomy.

## Conclusions

The decompressive laminectomy is a commonly performed surgery. Although there have been many technical advances, some facilities may not have access to the costlier surgical tools or the familiarity with them. Here, we describe the use of a handheld osteotome, which is widely available and at low cost, in performing a laminectomy. Our experiences with cadavers and a case study show that this technique appears to be safe and effective. The technique also has the potential to reduce the actual duration of laminectomy, especially in multi-level laminectomies. Future studies should investigate the feasibility of this technique along with the role of the exoscope.
